# American Public Health Association

**Published:** 1888-12

**Authors:** 


### AMERICAN PUBLIC HEALTH ASSOCIATION.

The sixteenth annual meeting of the Association was held in Milwaukee, November 21, 23, and 24. Dr. C. N. Hewitt, of Red Wing, Minnesota, presided.

The first report made was that of Major CHARLES SMART, of the United States Army, being the report of a special committee on "The Pollution of Water Supplies," insisting on the harmfulness of sewage in potable waters from whatever source derived, whether from wells or larger sources of supply—whether supplied to dwellings or populous cities, and however remote the source of the contamination might be, so long as the sewage could in any way reach the water supply. The report drew attention to the fact that the pollutions of the smaller sources of supply of water were recognized and remedied, whereas the larger sources, though more injurious, may be overlooked or longer disregarded, and instanced the contamination of the waters by the sewage of the city of Chicago as pumped into the canal from the river and conveyed to the towns along that canal, and considered the matter of purification of water in its passage. Although that report admits that aëration doubtless does measurably promote the oxidation of organic matter under such circumstances, it claimed that various bacteria and bacilli that are known causes of malarial and typhoid fevers and many epidemic diseases were propagated under such circumstances. It stated also that settling basins, such as are used at St. Louis, nor large filters, such as are used at Atlanta, remedy this evil; that it is neither the odor nor discoloration that are the index of the pollution and potability of any water supply. Those may be remedied so far as can be observed by the unaided eye, but only careful microscopical examination or chemical analysis can determine those points.

The report indorsed the verdict of English authorities that rivers which have received sewage, even though such has been purified before it is discharged, are not safe sources of potable water. The conclusion was that, reduced to its lowest and simplest terms, the question of water supply is this: "The raising of sufficient money to bring in the wholesome water, and the investment of the health officer with power to preserve the wholesome quality of the public supply, and to prevent the use of water from sources which are known to be unwholesome." He cited the advantages resulting from an establishment of State boards of health in Massachusetts, Illinois, and Minnesota, with the power conferred upon them to examine into and regulate the sources of water supply within those cities.

The annual address of President HEWITT was next delivered, which maintained that the cause of public sanitation needs intelligent, hearty, and constant interest in, and support from, all classes of people. The aim of the Association should be to secure the diffusion of the greatest amount of intelligence among all classes, to guard against those things which result in disease, and especially to protect the homes of the poor who have neither the intelligence nor the power to protect their own homes. He quoted the language of a former president, who said: "To secure for all most perfect action of body and mind, so long as it is consistent with the laws of life; to help make all growth more perfect, decay less rapid, life more vigorous, and death more remote, are the ends sought by public sanitation." He dwelt upon the advantages of detailed sanitary work at all times, and the advantage that it gave by systematic organization in case of the occurrence of epidemics, provided great care was exercised. He maintained that the highest efficiency of local boards, rural as well as urban, is of the first consequence, and that the true function of a National board should be, on the one hand, to facilitate and cement the independent helpfulness of State boards among each other; and, in the second place, to provide for a thorough international system of sanitary inspection in connection with our consular system.

A paper by Dr. BENJAMIN LEE, of Philadelphia, was read, giving a series of synoptical memoranda of observations made at various quarantine stations along the Atlantic coast, including that of New York, which, though expensive, had fallen into a dilapidated condition, and was hampered by antiquated and inadequate methods under a system of political spoils-grabbing and assessments, with an enormous income from fees and fines attached to office; that of Philadelphia, of abutting on a sewer-polluted marsh; that of Baltimore, situated too near the city, and lacking proper means for disinfection; that of Norfolk, Cape Charles, and Wilmington, described as sanitary sepulchres not even whited for appearance sake. From these observations he gave the following estimate of the system:

1. Want of uniformity in quarantine regulations, placing one port at a disadvantage, either commercially or sanitively, as compared with another.

2. Conflict of authority, owing to the methods of appointing officials.

3. Entire lack of appreciation on part of local legislatures, whether State or municipal, of the importance of the expenditure of considerable sums of money in order to render quarantines efficient and inoppressive.

4. Tendency on part of the local, civic, and sanitary authorities to limit their responsibility to the protection of their own city, reckless of the consequences which may ensue to inland communities if they permit infection, which circumstances render harmless to themselves, to pass unchallenged to the latter.

Dr. CROSBY GRAY, health officer of the city of Pittsburgh, Pennsylvania, presented a report on the contamination of that city's south side water supply by surface drainage, in which he gave the results of a very careful topographical, chemical, and biological examination into the general causes of a higher death rate on that side of the city than in other portions of the same city, and particularly regarding a severe epidemic of typhoid fever which occurred recently in the district described. His investigations demonstrated that the water supply drawn from the Monongahela River is being greatly and seriously polluted by sewage, factory refuse, and other nuisances, and that the epidemic referred to had been caused by the sudden down-wash through rainwater surface drainage of the excreta of typhoid patients through gullies above the in-take.

After an attempted estimate of money lost resulting from neglect of sanitary conditions, he claimed that this great waste might be stopped by the establishment of improved water service, drawing its supply from unpolluted sources, which would in the end prove an economical measure, even though the expenditure in securing such supply might be great.

Dr. H. B. BAKER, secretary of the State Board of Health of Michigan, advocated more methodical and thorough classification of vital statistics, based on scientific and instructive principles, in order that statisticians may be enabled to draw reliable and useful conclusions.

Dr. J. H. RAUCH, secretary of the State Board of Health of Illinois, read a paper on "Yellow Fever, Panics and Useless Quarantines, its Limitation by Temperature," in which he strongly urged the establishment of an efficient National board of health. He maintained that with the thermometer at 70° Fahr. or lower, there was no danger of the spread of such an epidemic of yellow fever as has recently occured in our Southern States and that much of the quarantining that had been done against them was unnecessary, and sometimes even cruel; that the importation of the disease should be provided against by the most careful, expert, consulary espionage, and kept out by rationally efficient coast quarantines.

He drew attention to the depressing influence of fear of individuals and panicky feeling existing in communities in time of epidemics, and maintained that frank and accurate statements of facts promptly and authoritatively made at such times do much to guard against the demoralized state which often accompanies such epidemics, and advocated that efficient and harmonious co-operation should prevail among the various State boards of health.

Dr. F. MONTIZAMBER, quarantine officer in the Province of Quebec, read a paper giving a detailed account of the equipment on land and water for the care of immigrants stationed on Grosse Isle, and appliances for disinfecting vessels. It was followed by a discussion as to whether sulphurous acid gas is better, generated by combustion in the hold, or forced into it by means of a fan.

Dr. L. N. SALOMON briefly described the New Orleans quarantine station, describing improvements in location and equipment, present and prospective.

Dr. S. H. DURGIN, quarantine officer at Boston, described the methods observed at that quarantine station.

Dr. S. S. KILVINGTON, president of the State Board of Health of Minnesota, read a paper, in which he described this as "the era of filth formation." He strongly advocated careful sanitary measures for every modern city. He also advocated the destruction of garbage of cities by cremation. He described the characteristics of the different crematories now in operation in this country.

In discussing the merits of the different forms of crematories now in use for the destruction of garbage, Health Commissioner DeWolf, of Chicago, stated that while he had believed in the practice of garbage destruction by fire, yet he had recently been converted to what he considers a more economic, yet sanitary, disposal of garbage by the Buffalo distillery crematory.

He was followed by Health Officer Clark, of Buffalo, who described that crematory. He stated that it is odorless in its working, and that the expense of running it was defrayed by lubricating oils which were extracted in the process, and that the result of the distillation furnished fuel for the running of the furnace, thus economizing in the use of coal.

Dr. J. COCHRAN, of Alabama, read a paper on the problems of yellow fever epidemics, in which he noted the fact that yellow fever has not obtained a permanent foothold in the United States, but believed that there was danger that it might do so in that part of Florida south of the frost line. Thus far the disease has been transferred from one locality to another by persons who have been in the infected district. He advanced the idea that cats, dogs, and other inferior animals, might be a cause of spread of the contagion, as has been claimed to have been done in times of scarlet fever and diphtheria epidemics. He drew attention to the greater danger of individuals to exposure in infected districts by night than by day. He believes that white people are more susceptible to an attack of scarlet fever than negroes, men more than women, adults more than children; that persons of dark complexion are more liable than those of fair complexion. He opposed the depopulation of infected cities, and claimed that the disease makes slow progress at first, sometimes being confined to a single house, and spoke of the advantages of care in isolating private residences, which often brings immunity to the occupants of residences thus isolated. He also opposed the formation of refugee camps.

Dr. A. N. BELL, with others, submitted evidence confirmatory of the impression that the disease is infectious rather than contagious; that the clothing of the individual may be a means of infecting other persons, but that the disease is not taken by one individual from another even by actual personal contact—facts which seemed to have been well established by previous experience.

Dr. BELL presented a report of the committee on sanitary and medical service of emigrant ships, which showed the great deficiences existing in that service.

The Lomb prize in hygienic dietetics was awarded to Mary J. Hinman.

Several papers were read by title. Some routine business was transacted, and the Association adjourned.

President for the following year, Professor H. A. Johnson, M. D., of Chicago, Illinois. Next place of meeting, Buffalo, New York.

